# The impacts of war on health, human rights, and the environment—an overview

**DOI:** 10.3389/fpubh.2025.1547784

**Published:** 2025-09-17

**Authors:** Barry S. Levy

**Affiliations:** Department of Public Health and Community Medicine, Tufts University School of Medicine, Boston, MA, United States

**Keywords:** war, human rights, environment, malnutrition, communicable diseases, noncommunicable diseases, maternal and infant disorders, mental and behavioral disorders

## Abstract

War adversely affects health, violates human rights, and contaminates the environment. Direct health impacts of war result mainly from explosive weapons. Indirect health impacts of war, which often occur more frequently than the direct impacts, are primarily due to damage to civilian infrastructure and forced displacement of populations. These indirect impacts include malnutrition, communicable diseases, exacerbation of noncommunicable diseases, maternal and infant disorders, and mental and behavioral disorders. In many wars, there is widespread violation of human rights and international humanitarian law. War and the preparation for war contaminate air, water, and land, increasing the risk of adverse health effects. Health professionals can play major roles in providing medical care to victims of war, documenting and performing research on the health impacts of war, educating and raising awareness, and advocating for policies and programs to prevent war and build sustainable peace.

## Introduction

War (armed conflict) has profound impacts on health, human rights, and the environment. War causes injuries, illnesses, disability, and premature mortality. War targets civilians and civilian infrastructure. War displaces populations. War violates human rights and international humanitarian law. War damages the socioeconomic environment and the fabric of everyday life. War destroys animal habitats and ecosystems. And war leads to interpersonal violence, self-directed violence, and collective violence, including more war.

This review aims to address the knowledge gap among health professionals about the health consequences of war on noncombatant civilians, a subject that is infrequently addressed in schools of medicine, public health, and other health professions. There have been a limited number of comprehensive reviews that cover the adverse health consequences of war. Many studies have focused narrowly on direct consequences, such as injuries caused by explosive weapons, while ignoring or understating the indirect health consequences of war, which are generally more widespread than the direct consequences.

This review is designed to provide a holistic understanding of the detrimental effects of war on health, human rights, and the environment, thereby demonstrating that these enormous costs of war outweigh any potential benefits of war. In addition, this review offers a framework for monitoring and assessing the multiple impacts of war, which can guide future analyses and inform public health responses.

## Background

The vast majority of armed conflicts occur within a single country. They are most often fought for control of government or resources and fueled by the availability of arms and intragroup animosity. During 2023, there were armed conflicts in 52 countries, most of which were internationalized civil wars, in which other countries provided military and/or financial support. Four of these were major armed conflicts, with more than 10,000 deaths in 2023, and 20 were high-intensity armed conflicts, with between 1,000 and 10,000 deaths in 2023 (1). While the wars in Ukraine and Gaza have received much media attention, most armed conflicts are out of sight and out of mind for the vast majority of people in Europe and the United States.

One-fourth of the world population lives in regions directly affected by armed conflict. In most wars, the majority of deaths are among noncombatant civilians, who are often purposely targeted as a strategy of war. In many wars, large numbers of people are forcibly displaced, most of them within their own countries as internally displaced persons and many others as refugees, who have crossed international borders. At the end of June 2024, there were approximately 123 million people who had been forcibly displaced: 72 million internally displaced persons, 44 million refugees, and 8 million asylum-seekers (2). The number of forcibly displaced people has tripled since 2012. The plight of internally displaced persons is generally far worse than that of refugees because of inadequate food, water, shelter, healthcare, and security.

## Adverse consequences of war

### Direct morbidity and mortality

Direct morbidity and mortality during war occur as a result of indiscriminate and targeted attacks, mainly with explosive weapons, ranging from bombs to improvised explosive devices to antipersonnel landmines. Especially vulnerable to targeted attacks are women and adolescent girls who, during war, generally have reduced security because of breakdown in societal protections and because of the absence of family members to protect them. War disrupts the fabric of everyday life, especially traumatic in cultures where families are dependent on each other.

Attacks on healthcare—in violation of international humanitarian law—have been perpetrated by government and opposition forces, external aggressors, and non-state actors. These attacks have involved shelling, bombing, and looting of clinics and hospitals and assaulting, arresting, kidnapping, torturing, and murdering health workers and their patients. In war zones in 2023, there were 2,562 reported incidents of violence against, or obstruction of, healthcare—a 25% increase from 2022. In these incidents, 487 health workers were killed, 445 were arrested, 240 were kidnapped, and many others were injured. In addition, these incidents resulted in loss of healthcare for many people (3).

### Indirect morbidity and mortality

Most morbidity and mortality during war occurs indirectly as a result of damage to civilian infrastructure and forced displacement of populations. Infrastructure damage includes damage to farms and food supply systems, water treatment plants and supply systems, healthcare and public health services, generation and supply of electric power, and communication and transportation networks.

Evidence indicates that, during war, indirect deaths occur much more frequently than direct deaths. A 2020 analysis, based on more than 1,100 armed conflicts between 1990 and 2017, estimated that indirect deaths due to war during this period totaled 29.4 million—approximately 1 million per year (4). In contrast, during this same 28-year period, the Uppsala Conflict Data Program recorded approximately 50,000 deaths per year—many fewer—in state-based armed conflicts globally (5). More recent data, for the 2021–2023 period, indicates that there were an annual average of 159,356 conflict-related fatalities due to violence (6).

The occurrence of more indirect than direct deaths during war is consistent with mortality studies in specific wars. For example, during the civil war in the Democratic Republic of the Congo, studies documented that about 95% of all deaths were not due to weapons, but rather the result of damage to civilian infrastructure (7). As another example, in the armed conflict in Darfur, Sudan, from 2004 to 2008, there were an estimated 300,000 excess deaths, more than four-fifths of which were not due to violence; many of these deaths were due to diarrheal diseases and other risk factors not directly related to violence, such as overcrowding, poor sanitation, and inadequate health services (8).

Indirect morbidity and mortality occur mainly in the following five categories: malnutrition, communicable diseases, noncommunicable diseases, maternal and infant disorders, and mental and behavioral disorders, as described in more detail below.

#### Malnutrition

Malnutrition is of special concern for children, whose physical and neurobehavioral development is adversely affected, and for pregnant and lactating women. During war, malnutrition arises and persists because of reduced production of food; damage to food storage facilities, transport infrastructure, and markets; delay and diversion of the food supply by corrupt government officials or military forces; restriction of food import because of embargoes or economic sanctions; inadequate humanitarian food assistance; unavailability or inaccessibility of health services; and various forms of discrimination.

Between 1990 and 2021, seven of the eight famines with 50,000 or more deaths were related to armed conflict (9). These famines occurred in Somalia (twice), Sudan (twice), Malawi, Uganda, and the Democratic Republic of the Congo (10).

In violation of international humanitarian law, food has increasingly been used as a weapon during war, causing mass starvation and famine, most severely affecting children, older people, and people with chronic medical conditions or disabilities. For example, during the Yemeni Civil War, food has frequently been used as a weapon; more than 400,000 children suffered from severe acute malnutrition, largely the result of air attacks that targeted infrastructure for food production and transport, and air and naval blockades that severely restricted food import (11).

#### Communicable diseases

Communicable diseases during war are primarily (a) bacterial and viral diarrheal diseases, such as cholera, and (b) highly contagious respiratory disorders, such as measles (with a high case-fatality rate among non-immunized children). For example, in conflict-affected northern Syria in 2017 and 2018, there were large outbreaks of measles with approximately 23,600 clinically suspected cases, compared to 3,193 cases reported throughout the country in the decade before the Syrian Civil War began (12). During war, diarrheal diseases occur primarily because of breakdowns in personal hygiene and sanitation and because of damage to water treatment plants and supply systems, resulting in consumption of water contaminated with microorganisms. For example, during the Yemeni Civil War, a large outbreak of cholera took place, with more than 1.2 million cases and more than 3,000 deaths in the first 6 months (13). During war, respiratory diseases can be easily transmitted because of crowding in bomb shelters, refugee camps, and elsewhere. The risk of other communicable diseases, such as malaria and leishmaniasis, during war results from their endemicity in war zones.

Contributing factors to the increased occurrence of communicable diseases include damage to healthcare facilities, injuries and deaths of health workers and weakened public health agencies, with limited resources for immunizations and for investigation and control of disease outbreaks. Another contributing factor is increased resistance to antibiotics. For example, multidrug-resistant tuberculosis (MDR TB) occurs more frequently during war. A nationwide survey in Somalia in 2011, after two decades of civil war, found that MDR TB was present in 5.2% of newly diagnosed TB patients and 40.8% of patients with previously treated tuberculosis—among the highest levels of drug resistance ever recorded in Africa and the Middle East (14).

#### Noncommunicable diseases

Exacerbations of noncommunicable diseases, such as cancer, cardiovascular disease, chronic lung disease, and diabetes mellitus, occur more frequently during war, mainly due to reduced access to medical care and routine medications. For example, surveys done by the World Health Organization in war-torn Ukraine in September and December 2022, found that half of the respondents reported at least one barrier to accessing healthcare and 22% in the first survey and 11% in the second survey were unable to receive medication that they needed (15). In addition, humanitarian aid organizations that provide assistance to victims of war generally do not give adequate attention to noncommunicable diseases.

When healthcare and medications for noncommunicable diseases are not available, the health risks of the affected population worsen. For example, people with cancer are at increased risk of complications and death; people with asthma are at increased risk of severe attacks; people with diabetes mellitus, especially those requiring insulin, are at increased risk of severe complications and death; and people with hypertension are at increased risk of myocardial infarction and stroke. It has been shown that treatment of elevated blood pressure can reduce ischemic heart disease by half and the incidence of stroke by about two-thirds (16). In addition, during war, preventive measures for noncommunicable diseases, such as screening for breast and colon cancer, are unlikely to be available.

#### Maternal and infant disorders

During war, pregnant women and their newborn infants face increased risks of morbidity and mortality due to inadequate prenatal, intrapartum, and postpartum care. Inadequate care arises from attacks on clinics and hospitals; injuries and deaths of physicians, nurses, and other healthcare workers; and reduced availability of basic medical supplies. Because of decreased access to prenatal care and treatment, pregnant women are at increased risks for pre-eclampsia, hemorrhage during delivery, and death. During war, there are also increased risks of premature birth, low birthweight, and infant mortality. A study found that, of the 15 countries with the highest rates of neonatal mortality (death during the first month of life), 14 have had chronic conflict or political instability (17). Maternal and infant health during war can be enhanced by improving access to and the quality of antenatal, intrapartum, and postpartum care and reproductive health services.

#### Mental and behavioral disorders

Mental and behavioral disorders increase during war, including posttraumatic stress disorder, depression, alcohol use disorder, drug abuse, and suicide (18). The impact of mental and behavioral disorders is especially profound on children, seriously affecting their social, intellectual, and emotional development. Often mental and behavioral disorders have lifelong, and even intergenerational, consequences. Many factors contribute to the increased occurrence of these disorders during war, including physical and sexual trauma, family separation, deaths of loved ones, damage to the environment, loss of employment and education, forced displacement, witnessing atrocities, and uncertainty about the future (19).

### Health impacts on military personnel

While this review focuses on the consequences of war to noncombatants, it is important to acknowledge the extensive impacts of war on military personnel, many of whom were noncombatant civilians before being forced to join the military. Combatants suffer extensive morbidity and mortality during war largely due to explosive weapons. Military personnel with nonfatal war-related injuries frequently develop infectious complications, long-term disabilities, and chronic pain. Combatants are at increased risk of developing posttraumatic stress disorder, depression, substance use, and suicide. On returning home after deployment, veterans face additional challenges to their mental and behavioral health, which often impact their families and communities.

### Violation of human rights and international humanitarian law

Principles for the protection of civilians focus on the justification for war, justified conduct during war, and justice after war. International humanitarian law includes three principles for justified conduct during war:

- *Distinction* discriminates between combatants, who during war are considered to be legitimate targets, and noncombatant civilians, who are not.
- *Necessary or minimal force* mandates that military personnel use the minimal amount of force necessary in order to attain legitimate military goals and objectives.
- *Proportionality* mandates that civilian harm and civilian property damage are not excessive compared with the military advantage anticipated by an attack on a legitimate military target.

These principles have frequently been violated in many recent wars, such as when noncombatant women have been sexually assaulted, noncombatant men have been summarily executed, children have been abused or kidnapped, people have been forcibly displaced, and civilian infrastructure has been attacked, resulting in deprivation of food, water, shelter, and healthcare.

### Inequities of war

The French philosopher Jean-Paul Sartre said, “When the rich wage war, it’s the poor who die.” In most wars, people who are already facing threats to their health and wellbeing and those who lack political power are disproportionately affected. War exacerbates pre-existing inequalities, such as discrimination against older people and people with disabilities, resulting in their being less able to flee and meet their daily needs (20).

### Impacts on the environment

War and the preparation for war adversely affect the physical environment. Explosions and fires contaminate the air with chemicals and particulate matter, contributing to acute and respiratory disorders. White phosphorus, used as an incendiary weapon, can cause not only severe burns, but also deforestation (21). Military forces intensively use fossil fuels, releasing greenhouse gases, which contribute to climate change. War and the preparation for war frequently contaminate surface water and groundwater with organic solvents and other toxic materials. Land is contaminated with defoliants and other toxic chemicals. For example, during the Vietnam War, Agent Orange (a defoliant containing 2,4-D, a possible carcinogen) destroyed mangrove forests. Bombs also destroyed mangrove forests, leaving huge craters, which filled with water and became breeding places for mosquitoes that transmit malaria.

In many wars, land has been contaminated by antipersonnel landmines and unexploded ordnance. There are an estimated 110 million landmines still present in at least 58 countries, posing risks, especially for children, who may be injured or killed as a result of touching, or even approaching, landmines. Globally, during each month, at least 1,000 people die or are maimed by landmines. Since the implementation of the Mine Ban Treaty in 1999, 94 of its 164 states parties have destroyed 55 million landmines in their stockpiles (22). It costs as little as $3 to produce a landmine, but at least $300 to remove one.

In addition, the environment has been contaminated with ionizing radiation and radioactive materials, primarily from the manufacture of nuclear weapons. And, as has occurred during Russia’s war in Ukraine, attacks on nuclear power stations represent an additional risk of population exposure to ionizing radiation (23).

### Diversion of resources

Diversion of human and financial resources for military purposes is a major concern. Global military expenditures, in 2023, were $2.44 trillion, more than 675 times the total budget of the United Nations. Annual military expenditures by the United States total $916 billion, more than the next nine countries combined: China, Russia, India, Saudi Arabia, the United Kingdom, Germany, Ukraine, France, and Japan (24). Between 2018 and 2023 (excluding 2021, when there was no change in spending because of the COVID-19 pandemic), there was an annual average increase of 4.2% in global military expenditures (24). Between 2014 and 2023, there was a substantial increase in the concentration of global military expenditures, with the share of spending by the United States and China combined increasing from 44 to 50% over this period of time, and the combined spending of the top 10 countries rising from 69 to 74% (25). Globally, national governments spent an average of 6.9% of their budgets for military purposes—$306 per person (26).

The diversion of resources for military purposes was illustrated by an analysis performed 20 years ago. It was estimated that the first $204 billion spent by the United States for the Iraq War could have decreased world hunger by 50% and provided globally, for 3 years (a) needed medicines for HIV/AIDS, (b) clean water and sanitation, and (c) immunization for all children in “developing countries” (27).

As U.S. President Dwight Eisenhower said in 1953: “Every gun that is made, every warship launched, every rocket signifies, in the final sense, a theft from those who hunger and are not fed, those who are cold and not clothed. This world in arms is not spending money alone. It is spending the sweat of its laborers, the genius of its scientists, the hopes of its children.” (28).

As a reflection of this diversion of resources, Costa Rica, which has had no military expenditures since 1948, ranks first in life expectancy in Central America and South America, and its standard of living is almost double that of other Central American countries, except Panama (29, 30). If other countries followed Costa Rica’s decision to disband their military forces, resources currently used for military expenditures could be used for societal benefit. As Indira Gandhi said: “Peace we want because there is another war to fight—against poverty, disease, and ignorance.”

## Exacerbating factors

### International trade in conventional weapons

The international arms trade increases the availability of conventional weapons in many countries, making them directly available to armies, militias, and insurgent groups, and indirectly to civilians—thereby increasing the likelihood of armed conflict and severe health consequences. The volume of international arms transfers during the past 15 years has remained about the same. The volume of these transfers during the 2009–2023 period was much higher than during the 1994–2008 period; however, it was about one-third lower than the volume of international arms transfers during the 1974–1988 period, when they peaked (31).

The Arms Trade Treaty, which entered into force in 2014, promotes accountability and transparency of arms transfers, including by banning arms transfers to countries that violate relevant international obligations or would use the arms for genocide, crimes against humanity, grave breaches of the 1949 Geneva Conventions, attacks against civilians or “civilian objects,” or other war crimes (32). However, the Treaty does not limit the amounts or types of arms that may be bought, sold, or possessed by countries. There remains much illicit transfer of arms, which contributes to sociopolitical instability, political repression, human rights violations, crime, and armed conflict (33).

International humanitarian law bans the use of weapons that do not distinguish between noncombatant civilians and combatants and those that cause unnecessary suffering. These weapons include chemical, biological, and nuclear weapons; antipersonnel landmines; and cluster munitions. In addition, the use of unmanned drones raises important ethical issues related to civilian injuries and deaths, the absence of direct consequences to drone operators, and targeted killings.

### Nuclear weapons

Nuclear weapons pose an existential threat to humankind. The United States detonated “atomic bombs” over Hiroshima and Nagasaki in 1945 at the end of World War II, causing more than 200,000 immediate and short-term deaths from heat, blast force, and ionizing radiation and eventually tens of thousands of cases of cancer and nonmalignant diseases. The development, production, and testing of nuclear weapons has caused adverse health, environmental, and socioeconomic impacts, disproportionately affecting Indigenous Peoples, minority group members, and other vulnerable populations.

There are approximately 12,000 nuclear weapons possessed by nine countries, about 88% by the United States and Russia (34). Most of them are much larger than the bombs that were dropped on Hiroshima and Nagasaki. Nuclear weapons could be launched by accident or due to misinterpretation or miscommunication (35). Even a small nuclear war could lower temperatures globally and cause widespread famine (36).

### Autonomous weapons systems and artificial intelligence

Autonomous weapons systems, which independently identify targets and destroy them without meaningful human control, might reduce civilian casualties and the number of soldiers on battlefields, but they might also lead to widespread killing and accidental escalation of conflicts (37, 38). In addition, it is immoral to give machines, operating without direct human control, the ability to determine who lives and who dies during war (39). Also of concern are decision-support systems based on artificial intelligence, in which computer systems perform tasks that normally require human intelligence; the accuracy of these systems depends on the validity of the algorithms and the information being fed into these systems.

## Discussion

### Documenting the health impacts of war

The Greek playwright Aeschylus wrote, “In war, truth is the first casualty.” As health professionals, we need to be committed to finding out the truth about the health impacts of war by overcoming challenges to achieving this goal, including:

- Difficulties in gathering information in war zones because of inadequate security and political instability
- Inaccurate reporting of morbidity and mortality by sources that have incentives to either overstate or underestimate these consequences of war
- Damage to systems for collecting and analyzing health data
- Displacement of populations
- Challenges in determining the magnitude of indirect health impacts
- Distant or remote health impacts of war
- Delayed impacts, including mental health problems and noncommunicable diseases with long latency periods, such as various forms of cancer
- Intergenerational psychosocial effects.

The three overall approaches for the recognition and assessment of the health consequences of war are rapid assessments, public health surveillance, and epidemiological studies.

Rapid assessments collect information from field observations, small-scale surveys, and interviews with affected individuals, government officials, community leaders, and other key informants. Information is obtained not only on morbidity and mortality, but also on vulnerable groups, violations of human rights, availability of food and safe water, hygiene and sanitation, shelter and security, and community organization. These assessments are critical for ensuring that appropriate humanitarian aid reaches people in need.

Public health surveillance obtains information from a wide variety of sources, including physicians and other health workers, clinics and hospitals, clinical laboratories, and death registers as well as government agencies, humanitarian aid organizations, journalists, and social media. Surveillance data, although incomplete, can identify significant trends and outbreaks, causes and risk factors for illnesses and injuries, and the needs of affected populations.

Epidemiological studies can provide valuable information on the nature, severity, and magnitude of health problems and their causes—and can identify opportunities for prevention. Epidemiological studies are used to inform the public as well as government and aid officials, guide implementation of health programs, and facilitate coordination among agencies and organizations. Prospective mortality studies can identify, confirm, and describe the circumstances of direct deaths as they occur, based on multiple sources, including death certificates, news reports, burial sites, and death-benefit programs. Retrospective mortality studies can yield estimates of previous deaths, such as by interviewing members of selected households. While performing research in war or postwar settings, epidemiologists need to rely on local partners, contingency plans, or unanticipated circumstances, understand local culture and political obstacles, and recognize that timely dissemination of results is critically important (40).

Forensic investigations can help to document torture and other human rights abuse during war and its aftermath. These investigations include physical examinations of torture survivors and the bodies of deceased persons, systematic interviews of witnesses, and other methods (41).

### Prevention

Each of the health impacts of war can be reduced by improved protection of civilians and civilian infrastructure and by improved medical care and humanitarian assistance. Noncombatant civilians can be better protected against assaults and injuries. Malnourished children can be provided adequate nutrition and necessary medical care. Communicable diseases can be prevented by immunizations, control of disease outbreaks, provision of safe water, and maintaining personal hygiene and sanitation. Mental health services can be improved. And people can be protected from harmful environmental exposures. But the only way to eliminate the health impacts of war is to eliminate war.

Approaches to the prevention of war include:

- Resolving disputes nonviolently by diplomacy, arms control, and measures to defuse conflicts and to prevent the spread of violence
- Addressing the root causes of war, such as militarism, socioeconomic inequities, ethnic and religious animosities, poor governance, and environmental stress
- Strengthening the infrastructure for peace, such as by rehabilitating nations and reintegrating people after conflict, establishing truth and reconciliation commissions, deploying international peacekeepers, ensuring the roles of women in the peace process, strengthening civil society, promoting the rule of law, ensuring citizen participation in decisions that affect their lives, and holding aggressors accountable (42).

### Reasons for hope

Despite the overwhelming impacts of armed conflict, there are reasons for hope. For example, although more than 250 rivers are shared by two or more countries, many disputes over shared water supplies have been resolved without violence. Globally, there is increased respect, protection, and fulfillment of human rights. Humanitarian assistance during conflict has been improved and made more systematic. Some international treaties have been extremely effective, such as the Chemical Weapons Convention, which has led to the almost total elimination of chemical weapons, and the Mine Ban Treaty, which has substantially reduced the number of antipersonnel landmines and the production of new landmines. And more people and organizations are working to prevent war and to promote peace.

### Roles of health professionals

Physicians and other health professionals can play important roles in minimizing the health consequences of war and in helping to prevent war. These roles include:

- Directly providing medical care and rehabilitation services, both during and after war, including services for refugees from war-affected countries and for military veterans.
- Documenting and performing research on the health impacts of war as well as their causes and preventive measures.
- Educating and raising awareness among health professionals, policymakers, and the general public about the health consequences of war.
- Advocating for policies and programs to minimize the consequences of war, prevent war, and build sustainable peace.

## Summary and a call to action

This review has highlighted the adverse consequences of war on health, human rights, and the environment. To highlight its most important points:

- Noncombatant civilians bear the burden of these adverse consequences.
- Indirect health impacts of war occur more frequently than the direct impacts, which are caused mainly by explosive weapons.
- The indirect impacts are primarily due to damage to civilian infrastructure and forced displacement of populations.
- These consequences of war, as well as war itself, can be prevented.

We health professionals need to play greater roles in addressing and preventing the consequences of war on health, human rights, and the environment. We need to better inform ourselves, our colleagues, policymakers, and the general public about these consequences. We need to be more effective in advocating for the protection of civilians during war and for the prevention of war. And we need to ensure that schools of medicine, public health, and other health professions promote education and research on war—the greatest threat to the health of humankind.
